# Variable Food-Specific IgG Antibody Levels in Healthy and Symptomatic Chinese Adults

**DOI:** 10.1371/journal.pone.0053612

**Published:** 2013-01-03

**Authors:** Qiang Zeng, Sheng-Yong Dong, Liu-Xin Wu, Hong Li, Zhi-Jian Sun, Jing-Bo Li, Hong-Xia Jiang, Zhi-Heng Chen, Qi-Bin Wang, Wei-Wei Chen

**Affiliations:** 1 International Medical Center, Chinese PLA General Hospital, Beijing, China; 2 The Second Laboratory, PLA Air Force Institute of Aviation Medicine, Beijing, China; 3 Health Management Center, Fujian Provincial Hospital, Fuzhou, Fujian, China; 4 Health Management Center, Shandong Provincial Hospital, Jinan, Shandong, China; 5 Health Management Center, Southwest Hospital, The Third Military Medical University, Chongqing, China; 6 Health Management Center, Xiangya Hospital, Central South University, Changsha, Hunan, China; 7 Health Management Center, The Third Xiangya Hospital of Central South University, Changsha, Hunan, China; 8 Health Management Center, Shandong Electric Power Center Hospital, Jinan, Shandong, China; 9 Treatment and Research Center for Infectious Diseases, 302 Hospital of PLA, Beijing, China; Ludwig-Maximilians-University Munich, Germany

## Abstract

**Background:**

The presence of food-specific IgG antibodies in human serum may be useful for diagnosis of adverse food reactions. However, the clinical utility of tesing for such antibodies remains very controversial. The aim of this study was to evaluate the serum levels and population distribution of food-specific IgGs and their association with chronic symptoms in a large-scale Chinese population.

**Methodology/Principal Findings:**

A total of 21305 adult participants from different regions of China had 14 type of food-specific serum IgG antibodies that were measured by enzyme-linked immunosorbent assay. Amongthese, 5,394 participants were randomly chosen to complete follow-up questionnaire surveys on their dietary characteristics and chronic symptoms. The concentrations of food-specific IgGs against 14 foods ranged from a median (interquartile range) of 7.3 (3.8, 12.6) U/mL of pork-specfic IgG to 42.3 (28.8, 60.2) U/mL of crab-specific IgG. The concentration of food-specific IgGs was closely related to gender; after adjustment for region and age, women had higher concentrations of food-specific IgGs against all of the 14 foods except chicken (regression coefficient (95% CI): 0.01 (−0.003, 0.023); *P* = 0.129) and corn (0.002 (−0.013, 0.016); *P = *0.825). Similar results were also found in the relationship of geographic region to the food-specific IgG concentrations for the 14 foods. Chronic symptoms were negatively associated with the concentrations of a few food-specific IgGs, and were positively associated with the concentrations of other food-specific IgGs.

**Conclusions:**

The levels of food-specific IgGs were variable both in healthy and in symptomatic Chinese adults. These findings raise awareness that demographic factors, the type of food and specific chronic symptoms should be considered before food elimination treatment based on IgG testing in patients with chronic symptoms is used in clinical practice.

## Introduction

The prevalence of migraine, asthma, allergic dermatitis and irritable bowel syndrome has been continuously increasing. Etiological studies suggest that these diseases may be related to adverse food reactions (food hypersensitivity) [1], [2], [3], [4], [5]. According to a recent report of an expert panel sponsored by the U.S. National Institute of Allergy and Infectious Diseases [6], adverse food reactions include food allergies and food intolerances, and the gold standard for the diagnosis of adverse food reactions remains the double-blind, placebo-controlled food challenge (DBPCFC). However, DBPCFC is difficult to use in large population studies due to the tedious, inconvenient, and costly procedures involved [7]. Other diagnostic tests such as skin prick tests, serum food-specific IgE tests, and atopy patch tests lack specificity and may lead to the unnecessary exclusion of foods from the patient’s diet [6].

Previous studies have found that the levels of food-specific IgGs and IgG subclasses in blood serum were significantly higher in individuals with food hypersensitivity [8], [9], [10], and that IgG-mediated immunologic responses may play an important role in the pathogenesis of adverse food reactions [11], [12], [13]. However, other studies indicate that the serum level of food-specific IgGs may also be elevated in healthy individuals, suggesting that the elevation of food-specific IgGs and IgG subclasses may merely reflect the presence of immunological tolerance to food-specific antigens, i.e., that it may only reflect a history of exposure to a certain food [14], [15]. Therefore, it was determined that food-specific IgGs and IgG subclasses lack diagnostic value for food allergy/intolerance [15], [16], [17].

Although the diagnostic utility of the presence of food-specific IgGs in patients with adverse food reactions remain controversial, serum food-specific IgG tests have been used in patients with chronic symptoms because of their convenience [18], [19]. In addition, selective food elimination based on the results of food-specific IgG testing has been suggested to be effective in the treatment of irritable bowel syndrome and migraine [4], [5]. However, the distribution of food-specific IgG concentrations, and the relationship of food-specific IgGs to chronic symptoms has not been reported in any large-scale population studies [18]. The aim of this study was to investigate the factors related to the presence of food-specific IgGs and their association with diet characteristics and chronic symptoms in a large adult population from various regions of China.

## Materials and Methods

### Study Population

The study population was recruited as patients presented to the clinic and underwent routine physical examination in general hospitals in 23 provinces in China (Figure S1), beginning in August 2008. Participation in the study was voluntary. Individuals aged 18–75 years were randomly selected using a single-blind method;, patients who were pregnancy or had a history of anaphylactic shock, secondary chronic symptoms caused by other known cardiovascular diseases, chronic kidney diseases or organic intestinal diseases were excluded. The concentrations of serum IgGs that reacted with 14 foods that are commonly consumed in China were measured in these individuals. By October 2010, a total of 21305 participants were included after excluding those with incomplete data. From this population, 6000 subjects were randomly selected and contacted for a questionnaire survey. Informed written consent was obtained from all participants, and the study protocol was approved by the Institution Review Board of the Chinese PLA General Hospital, Beijing, China.

### Questionnaire Design

A questionnaire was designed with questions adapted to Chinese adults. The questions included consumption frequencies of 14 common foods: beef, chicken, codfish, corn, crab, eggs, mushrooms, cow’s milk, pork, rice, shrimp, soybeans, tomatoes and wheat. Regular consumption referred to a consumption frequency of ≥3 times/week, and rare consumption referred to a consumption frequency ≤ one time/3 months; moderate consumption was defined as a consumption frequency that fell between these two extremes. The questionnaire also included information about perceived connections between ingestion of specific foods and chronic symptoms, such as gastrointestinal symptoms (abdominal pain, diarrhea or nausea), eczema, urticaria, rhinitis, asthma, arthritis, migraine headaches or other chronic symptoms (chest pain, or heart discomfort).

### Food-specific Serum IgG Assays

Participants’ sera were collected and sent, with only numerical identifiers, to authorized hospital laboratories, where enzyme-linked immunosorbent assays (ELISA) were performed to measure the concentrations of 14 different food-specific IgGs using a commercially available kit (Biomerica, Inc. USA). This assay has been described in a previous study [20]. Briefly, the serum samples were diluted 100-fold, and added to reaction wells coated with one of 14 food-specific allergens. After the plate was sealed, incubated at room temperature for 1 hour, washed and patted dry, anti-human IgG antibody conjugated to horseradish peroxidase was added to the wells. After another incubation at room temperature for 30 minutes, washing and patting dry, 100 µl of a substrate mixture consisting of equal proportions of 3,3′,5,5′-tetramethylbenzidine and hydrogenperoxide was added. The reaction was stopped after 10 minutes, and absorbance was measured at 450 nm. The concentrations of food-specific IgGs (U/mL; 1 U = 1.47 ng) were calculated using the standard curves of 14 types of food-specific IgGs provided by the manufacture. The detection limit was 0.01 U/mL. All authorized laboratories were asked to satisfy several standards of quality assurance for the determinations of these 14 types of food-specific IgGs. The quality assurance standards were as follows: the optical density of the negative controls should be <0.2, the concentrations of IgG in the positive quality controls should be >100 U/mL and the optical density in 50 U/mL, 100 U/mL, 200 U/mL and 400 U/mL standard wells should be 1.2 times more than in negative 50 U/mL, 100 U/mL and 200 U/mL standard wells, respectively. For the great majority of clinically relevant allergens, no standardized reference allergens are available for comparisons between methods. To ensure the reproducibility and accuracy of the ELISA kit assays, each serum sample was tested in duplicate parallel tests, all ELISA kits were required to be used within 10 days of their delivery from the factory, and all testing was performed using an automated spotting system that was supervised by well-trained medical laboratory technologists who were blinded to the clinical data. On average, the intra- and inter-assay coefficients of variant were 2.7% and 5.3%, respectively, in the Chinese PLA General Hospital laboratory, and 8.5% and 12.1% in other authorized laboratories. Before the study, all participating laboratories calibrated food-specific IgG measurements according to the quality control results distributed from the Chinese PLA General Hospital laboratory.

### Statistical Analysis

All data were entered into an Epidata 3.0 database through double-blinded entry, followed by automatic check and verification. Continuous variables are reported as median values with the corresponding 25th and 75th percentiles. Discrete variables are summarized in terms of frequencies and percentages. Because the data demonstrated a skewed distribution, serum concentrations of food-specific IgGs were logarithmically transformed; undetectable specific IgG concentrations (<0.01 U/mL) were considered 0.005 U/mL. Maximum likelihood estimation was performed to evaluate the means and standard deviations of food-specific IgG concentrations using R 2.15.2. To visualize the distribution of food-specific IgG concentrations in all subjects and demographic subgroups (sex, geographic region and age group), histograms or line graphs were generated, and one-way analysis of variance (ANOVA) and post-hoc Tukey tests were used for comparison of the resultings curves. *P*-values for trends were calculated when age group-related differences in food-specific IgG concentrations were examined. Pearson correlation was performed to analyze the relationship of food-specific IgG concentrations among the 14 foods. Differences in food consumption frequencies in demographic subgroups were tested using the Pearson chi-square test and *P* values for trends were calculated when differences in the food consumption frequencies of different age group were examined. Differences in food-specific IgG concentrations between subjects with chronic symptoms and subjects without chronic symptoms were also examined with ANOVA. Multivariate linear regression models were applied to evaluate the association of food-specific IgG concentrations of each of the 14 foods with demographic factors and food consumption frequencies. Logistic regression models were performed to analyze the association of chronic symptoms with food-specific IgG concentrations of the 14 foods, and odd ratios (OR) and 95% CI were calculated. All statistical analyses were performed with SPSS 10.0 (SPSS Inc., Chicago, IL, USA). Two-tailed P values <0.05 were considered significant.

## Results

### Study Population Characteristics

Of the 21305 subjects (mean age: 46.63±10.52 years; 13,426 men and 7,879 women), most (74.0%) were northern Chinese. Questionnaire replies were received from 5394 individuals (89.9% of the randomly selected subjects); of the 5394 individuals who responded to the questionnaire, 32.2% were women and 77.2% were northern Chinese. In the 2353 subjects who reported having chronic symptoms, the most common chronic symptoms were gastrointestinal symptoms (23.6%), rhinitis (11.8%) and migraine (9.5%) (Table 1).

**Table 1:** Baseline characteristics of the study population.

	Overall subjects, n = 21305 Number (%)	Subjects with questionnaire survey, n = 5394 Number (%)
Women	7879 (37.0)	1735 (32.2)
Age (year)		
18–34	1585 (7.4)	462 (8.6)
35–44	7526 (35.3)	1751 (32.5)
45–54	8268 (38.8)	2025 (37.5)
55–64	2849 (13.4)	733 (13.6)
≥65	1077 (5.1)	423 (7.8)
Region		
South China	5547 (26.0)	1231 (22.8)
North China	15758 (74.0)	4163 (77.2)
Chronic symptoms		
Gastrointestinal	–-	1274 (23.6)
Rhinitis	–-	636 (11.8)
Migraine	–-	511 (9.5)
Arthritis	–-	392 (7.3)
Eczema	–-	310 (5.7)
Urticaria	–-	259 (4.8)
Asthma	–-	153 (2.8)
Other	–-	721 (13.4)

### Distribution of Food-specific IgG Concentrations

**Figure 1:**
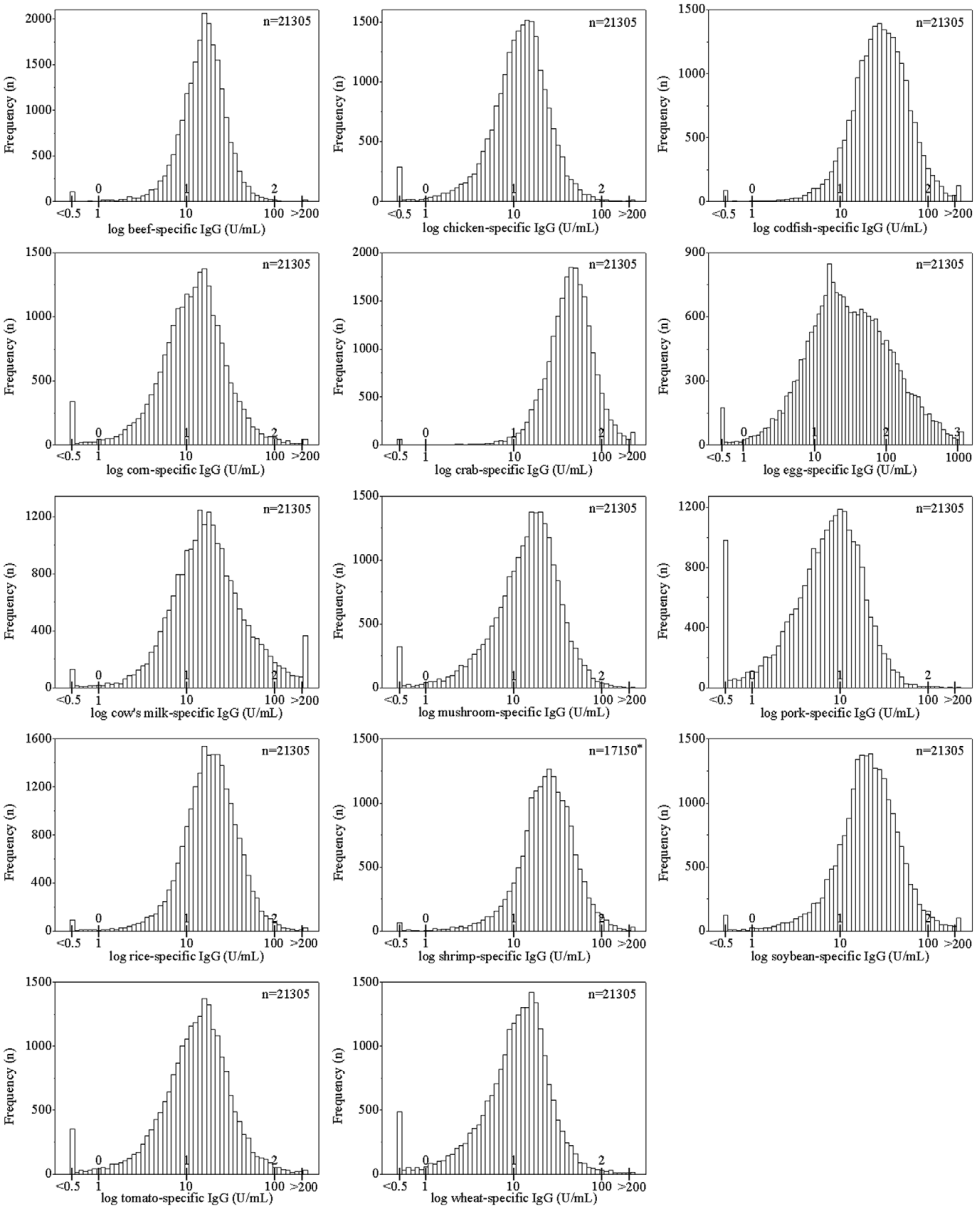
Distribution of serum food-specific IgG concentrations for 14 foods. ^*^The serum concentration of shrimp-specific IgG was recorded as the original value in 17150 subjects and has been classified as negative or positive in remaining 4155 subjects according to the directions provided by the manufacturer of the test kit.

The distributions of the serum concentrations of food-specific IgG against the 14 foods tested varied significantly (Figure 1). The log-distributed curves for food-specific IgG concentrations of crab, codfish, egg and shrimp are shifed slightly to the right; the medians (interquartile range) of the IgG concentrations against these foods were 42.3 (28.8, 60.2) U/mL, 27.5 (16.9, 44.2) U/mL, 26.6 (11.4, 72.5) U/mL and 22.8 (14.9, 35.2) U/mL, respectively. For pork-specific IgG, the log-distributed curve is shifted slightly to the left; the median and interquartile range of the concentration of pork-specific IgG is 7.3 and 3.8 to 12.6 U/mL. The means and standard deviations of log-transformed food-specific IgG concentrations that were calculated using maximum likelihood estimation are shown in Table S1.

The distribution of food-specific IgG concentration against egg varied more than for other foods. For example, a total of 61 subjects had levels of egg-specific IgG above 1000 U/mL, and the proportion of subjects who had egg-specific IgG levels above 200 U/mL was 8.4%, the highest od the 14 foods. The proportion of subjects who had the egg-specific IgG levels less than 10 U/mL was 21.7%; this level was higher than for other foods against which subjects had similar IgG levels, such as codfish (only 8.8% of the subjects had codfish-specific IgG levels that were less than 10 U/mL). Interestingly, the food-specific IgG levels against the 14 foods tested were all correlated with each other. The strongest relationship between food-specific IgG levels were found for beef and chicken (correlation coefficient, 0.691; *P*<0.01); the next strongest was for codfish and crab (correlation coefficient, 0.561; *P*<0.01) (Table S2).

### Relationship of Demographic Factors and Food-specific IgG Concentrations

Compared to the log-distributed curves of the frequencies of food-specific IgG concentrations in men, the log-distributed curves of the frequencies of food-specific IgG concentrations in women were shifted to the right for 10 of the 14 foods; the exceptions were chicken (median (interquartile) men vs. women: 11.5 (7.1, 17.3) U/mL vs. 11.8 (7.4, 17.9) U/mL; *P = *0.052), corn (men vs. women: 11.3 (6.5, 18.5) U/mL vs. 12.0 (6.9, 19.0) U/mL; *P = *0.378), mushrooms (men vs. women: 14.5 (8.1, 22.4) U/mL vs. 14.9 (8.1, 23.9) U/mL; *P = *0.065) and pork (men vs. women: 7.3 (3.8, 12.4) U/mL vs. 7.4 (3.8, 12.8) U/mL; *P* = 0.432). When the subjects were grouped according to sex, the greatest difference in food-specific IgG concentrations between men and women was found for egg (men vs. women: 22.4 (10.3, 61.2) U/mL vs. 36.8 (14.2, 93.6) U/mL; *P*<0.001); the next greatest differences were for cow’s milk (men vs. women: 14.9 (8.5, 26.2) U/mL vs. 18.2 (10.1, 34.3) U/mL; *P*<0.001) and soybean (men vs. women: 18.7 (11.7, 30.1) U/mL vs. 22.4 (13.9, 36.8) U/mL; *P*<0.001) (Figure S2).

Region subgroup histograms also showed that the log-distributed curves for almost all of the 14 foods shifted slightly for South Chinese compared to North Chinese subjects; the exceptions were chicken (south vs. north: 10.7 (7.1, 16.4) U/mL vs. 11.9 (7.3, 17.9) U/mL; *P = *0.333) and soybean (south vs. north: 18.7 (11.5, 30.8) U/mL vs. 20.5 (12.8, 33.0) U/mL; *P = *0.062) (Figure S3). Compared to subjects in North China, subjects in South China had higher mean concentrations of beef-specific IgG (south vs. north: 16.3 (11.6, 22.8) U/mL vs. 14.6 (9.9, 20.1) U/mL; *P*<0.001), corn-specific IgG (south vs. north: 11.7 (6.9, 19.7) U/mL vs. 11.5 (6.5, 18.3) U/mL; *P*<0.001) and shrimp-specific IgG (south vs. north: 24.9 (16.5, 37.6) U/mL vs. 22.6 (14.8, 35.0) U/mL; *P*<0.001). The mean concentrations of food-specific IgG against the other 9 foods were higher in North China than in South China, especially mushroom (south vs. north: 9.9 (5.1, 18.5) U/mL vs. 15.9 (9.6, 24.1) U/mL; *P*<0.001), pork (south vs. north: 4.8 (2.4, 9.1) U/mL vs. 8.3 (4.5, 13.6) U/mL; *P*<0.001) and wheat (south vs. north: 7.8 (4.1, 13.9) U/mL vs. 12.3 (7.3, 18.3) U/mL; *P*<0.001).

With increasing age of the subjects, the mean concentrations of food-specific IgG for almost all of the 14 foods increased. The exceptions were corn (11.9 (6.6, 19.4) U/mL, 11.4 (6.5, 18.7) U/mL, 11.3 (6.6, 18.3) U/mL, 11.8 (6.7, 18.5) U/mL and 13.2 (7.9, 21.7) U/mL for 20–34, 35–44, 45–54, 55–64 and ≥65 years age group, respectively; *P* for trend = 0.092) and wheat (12.1 (6.5, 20.0) U/mL, 10.5 (5.6, 17.2) U/mL, 10.9 (6.2, 17.3) U/mL, 11.6 (6.8, 17.5) U/mL and 13.4 (8.0, 18.8) U/mL for 20–34, 35–44, 45–54, 55–64 and ≥65 years age groups, respectively; *P* for trend = 0.115). Although food-specific IgG concentrations for the other 12 foods showed an increasing trend with age, the concentrations of egg-specific IgG and cow’s milk-specific IgG were significantly higher in the 18–34 years age group than in the 35–44, 45–54 and 55–64 years age groups (Figure S4).

**Figure 2:**
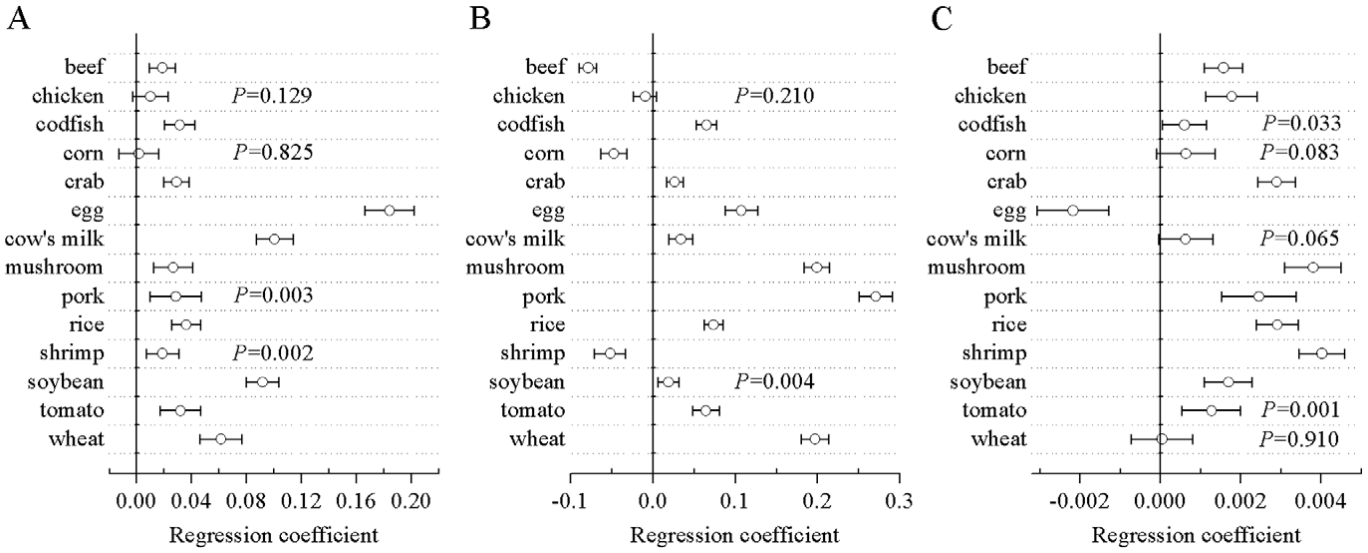
Association of demographic subgroups with serum food-specific IgG concentrations for 14 foods. (A) sex, (B) geographic region, (C) age. Regression coefficients and *P* values were derived from multivariate linear regression models that set sex (men, 0; women, 1), geographic region (South China, 0; North China, 1) and age as independent variables and the logarithmically transformed serum food-specific IgG concentrations for 14 foods as dependent variables. The food items for which *P* values are not shown yielded mean *P* values less than 0.001.

Multivariate analysis showed that women had higher food-specific IgG concentrations than men against all of the 14 foods except chicken (regression coefficient (95% CI): 0.01 (−0.003, 0.023); *P* = 0.129) and corn (0.002 (−0.013, 0.016); *P = *0.825) after adjustment for region and age. Of the other 12 foods, egg-specific IgG concentration showed the strongest relationship to sex (0.184 (0.166, 0.202); *P*<0.001). After adjustment for sex and age, subjects in South China had higher concentrations of beef-specific IgG (−0.079 (−0.089, −0.068); *P*<0.001), corn-specific IgG (−0.047 (−0.063, −0.031); *P*<0.001) and shrimp-specific IgG (−0.052 (−0.071, −0.033); *P*<0.001) than subjects in North China, whereas they had similar chicken-specific IgG concentration (−0.009 (−0.023, 0.005); *P* = 0.210) as subjects in North China; food-specific IgG concentrations against the other 11 foods were higher in subjects in North China. After adjustment for sex and region, age also showed strong relationship with to the food-specific IgG concentrations against all foods except corn (0.001 (0, 0.001); *P* = 0.083), cow’s milk (0.001 (0, 0.001); *P* = 0.065) and wheat (0 (−0.001, 0.001); *P* = 0.910). Age was inversely associated with egg-specific IgG concentration (−0.002 (−0.003, −0.001); *P*<0.001) and positively associated with food-specific IgG concentrations for the other 10 foods (Figure 2).

### Relationship between Consumption Frequency and Food-specific IgG

Of the 5394 subjects who responded to the questionnaire, the majority regularly consumed (≥3 times/week) rice and pork (83.8% and 70.1%, respectively); a minority of subjects regularly consumed shrimp and crab (15.4% and 6.9%, respectively). Men showed higher diet frequencies of codfish and meat, while more women regularly consumed cow’s milk and non-meat foods. The proportions of subjects who regularly consumed rice, crab, shrimp and codfish were higher in South than in North China, while the proportion of subjects who regularly consumed wheat was higher in North China population (69.7% vs. 35.4%, respectively; *P*<0.001).Shrimp, chicken, beef and pork were consumed most frequently by the 18–34 yearold age group, and the consumption of egg, cow’s milk, soybeans, corn, tomatoes and wheat increased with age (*P* for trend <0.001) (Table S3).

**Figure 3:**
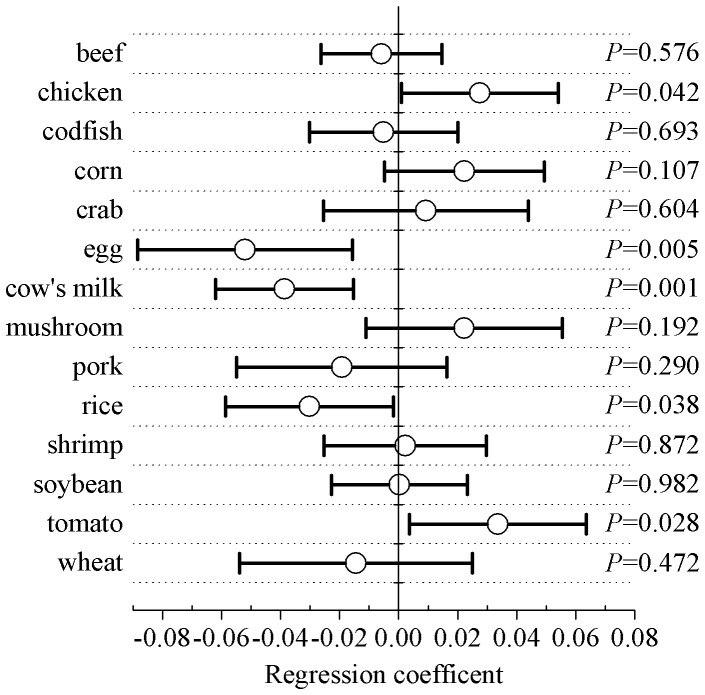
Association of various dietary frequencies of 14 foods with corresponding serum food-specific IgG concentrations. Regression coefficients and *P* values were derived from multivariate linear regression models in which the dietary frequencies of 14 foods (rare, 0; moderate, 1; regular, 2) were used as independent variables and the corresponding and logarithmically transformed serum food-specific IgG concentrations for 14 foods were the dependent variables. Demographic factorsincluding sex, region and age were adjusted for in these models.

After adjustment for sex, region and age, the diet frequencies of 9 of the tested foods were not significantly associated with the corresponding food-specific serum IgG concentrations. Dietary consumption frequencies of egg, cow’s milk and rice were inversely associated with egg-specific IgG (regression coefficient (95%CI): −0.052 (−0.088, −0.016); *P* = 0.005), cow’s milk-specific IgG (−0.039 (−0.062, −0.015); *P* = 0.001) and rice-specific IgG concentrations (−0.03 (−0.059, −0.002); *P* = 0.038), respectively; whereas, the dietary consumption frequencies of chicken and tomato were positively associated with chicken-specific IgG (0.028 (0.001, 0.054); *P* = 0.042) and tomato-specific IgG concentrations (0.034 (0.004, 0.064); *P* = 0.028), respectively (Figure 3).

### Relationship between Food-specific IgGs and Chronic Symptoms

Compared to subjects who reported having no gastrointestinal symptoms, subjects who reported having chronic symptoms had higher concentrations of codfish-specific IgG (median (interquartile) gastrointestinal symptom, yes vs. no: 23.8 (14.3, 45.1) U/mL vs. 20.9 (14.4, 37.9) U/mL; *P* = 0.013) and rice-specific IgG (16.7 (9.4, 28.0) U/mL vs. 15.4 (10.9, 22.0) U/mL; *P* = 0.032); and lower concentrations of mushroom-specific IgG (8.9 (4.3, 16.0) U/mL vs. 12.0 (6.5, 16.9) U/mL; *P* = 0.026), tomato-specific IgG (10.1(5.1, 17.7) U/mL vs. 13.1 (7.2, 18.4) U/mL; *P* = 0.018) and wheat-specific IgG (6.8 (3.0, 13.3) U/mL vs. 9.9 (4.6, 15.7) U/mL; *P* = 0.010). Subjects who reported having migraine also had lower concentrations of mushroom-specific IgG (8.4 (4.1, 15.7) U/mL vs. 11.8 (6.2, 16.9) U/mL; *P* = 0.007) and tomato-specific IgG (9.8 (4.6, 16.2) U/mL vs. 13.0 (7.0, 18.5) U/mL; *P* = 0.003) than subjects who reported having no migraine. Subjects who reported having eczema had higher concentrations of crab-specific IgG (44.8 (24.8, 66.5) U/mL vs. 37.3 (21.4, 58.5) U/mL; *P* = 0.038) and rice-specific IgG (17.0 (11.7, 27.2) U/mL vs. 15.5 (10.5, 22.9) U/mL; *P* = 0.009) than subjects who reported having no eczema. Rice-specific IgG concentration was higher in subjects who reported having urticaria than in subjects who reported having no urticaria (20.9 (13.0, 31.2) U/mL vs. 15.4 (10.5, 22.7) U/mL; *P* = 0.006); whereas, cow’s milk-specific IgG concentration was lower in subjects who reported having asthma than in subjects who reported having no asthma (14.2 (6.6, 23.7) U/mL vs. 15.3 (9.0, 27.7) U/mL; *P* = 0.042). The concentrations of food-specific IgG for cow’s milk, pork and wheat were also lower in subjects who reported having other symptoms than in those who reported having no other symptoms (Figure S5).

**Figure 4:**
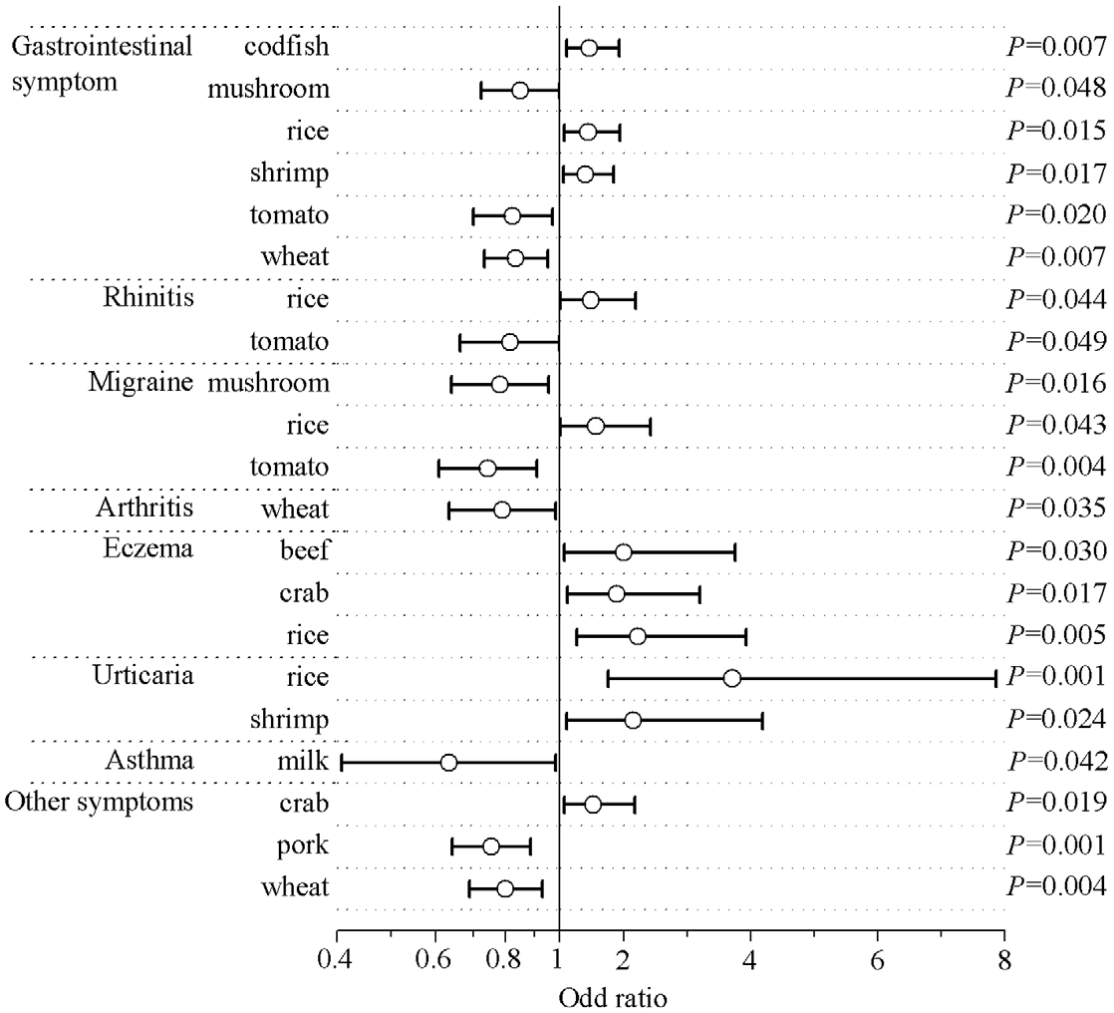
Association of chronic symptoms with food-specific IgG concentrations for 14 foods. Only the foods for which food-specific IgG levels were significantly associated with certain chronic symptoms (*P*<0.05) are listed**.** Odds ratios and *P* values were derived from logistic regression models that set logarithmically transformed serum food-specific IgG concentrations for the 14 foods as the independent variables and the presence of various chronic symptoms (No, 0; Yes, 1) as the dependent variables. Demographic factors including sex, region and age were adjusted for in these models.

The association of chronic symptoms with serum levels of specific IgGs against the 14 foods tested is shown in Figure 4. After adjustment for sex, region and age, gastrointestinal symptom were inversely associated with mushroom-specifi (OR (95% CI): 0.85 (0.72, 0.99); *P* = 0.048), tomato-specific (0.82 (0.70, 0.97); *P* = 0.020) and wheat-specific (0.84 (0.73, 0.95); *P* = 0.007) IgG levels; and positively associated with codfish-specific (1.47 (1.11, 1.94); *P* = 0.007), rice-specific (1.45 (1.08, 1.95); *P* = 0.015) and shrimp-specific (1.40 (1.06, 1.85); *P* = 0.017) IgG levels. Rhinitis was inversely associated with the serum concentration of tomato-specific IgG (0.82 (0.66, 0.99); *P* = 0.049) and positively associated with the concentration of rice-specific IgG (1.49 (1.01, 2.19); *P* = 0.044). Migraine was inversely associated with mushroom-specific (0.78 (0.64, 0.95); *P* = 0.016) and tomato-specific IgG levels (0.74 (0.61, 0.91); *P* = 0.004) and positively associated with rice-specific IgG levels (1.57 (1.01, 2.43); *P* = 0.043). Arthritis was inversely associated with wheat-specific IgG levels (079 (0.63, 0.98); *P* = 0.035). Eczema was positively associated with food-specific concentrations of beef-specific (2.01 (1.07, 3.75); *P* = 0.030), crab-specific (1.90 (1.12, 3.20); *P* = 0.017) and rice-specific (2.23 (1.27, 3.93); *P* = 0.005) IgG levels. Urticaria was positively associated with rice-specific (3.71 (1.76, 7.86); *P* = 0.001) and shrimp-specific (2.15 (1.11, 4.18); *P* = 0.024) IgG levels. Asthma was inversely associated with milk-specific IgG levels (0.63 (0.41, 0.98); *P* = 0.042). Other symptoms were inversely associated with pork-specific (075 (0.64, 0.89); *P* = 0.001) and wheat-specific (0.80 (0.69, 0.93); *P* = 0.004) IgG levels and positively associated with crab-specific IgG levels (1.53 (1.07, 2.17); *P* = 0.019).

## Discussion

Although commercial laboratories worldwide are currently offering broad-scale = testings for serum levels of food-specific IgGs to the public, to our knowledge, the study described here is the first representative cross-sectional study to address the distribution of food-specific IgGs and its relationship to chronic symptoms in a large population. Previous studies have either involved \ research on the potential involvement of various IgG subclasses in food allergy/intolerance [9], [21] or have addressed food elimination based on IgG testing [4], [5].

Although testing for the presence of food-specific IgGs has been regarded as a potential tool for the diagnosis of food allergy/intolerance, it’s the accuracy and clinical utility of such testing remain unclear. In our study, serum concentrations of food-specific IgGs against the 14 foods tested varied widely. Different foods have different proteins and components, and this may induce different immune reactions and lead to the presence of variable amounts of food-specific IgG. The completeness of extraction of different food proteins and other components of the 14 foods tested and the relative coupling efficiency of different extracted food components/antigens to the ELISA plate would also be expected to influence the accuracy of measurement of serum concentrations of food-specific IgGs. Although the levels of food-specific IgG against the 14 tested foods were significantly different, the concentrations of all 14 food-specific IgGs, especially IgGs directed against beef and chicken, were correlated with one another. Similar results were reported by Kanagawa et al [22] in an analysis of food antigens in 6549 subjects; the results of that study suggested that the appearance of food antigens clusters may be a result of immune cross-reaction between similar food antigens [6].

In our study, the distribution of food-specific IgG levels varied as a function of demographic subgroups (sex, region and age). Women had higher concentrations offood-specific IgGs against almost all of the tested foods than men. It has been consistently reported that the prevalence of food allergy/intolerance is significantly higher in women than in men [23], [24], [25]. In childhood, boys suffer from asthma, urticaria, anaphylaxis and food allergies to eggplant and peanuts more frequently than girls, while the sex difference is reversed after puberty [26], [27], [28], [29]. Studies suggest that some female sex steroids are pro-inflammatory and increase the susceptibility of atopy, while testosterone is a powerful inhibitor of histamine and known to inhibit mast cell degranulation [26], [30]. Moreover, for the first time, our study demonstrated that the distribution of food-specific IgGs against almost all of the 14 foods tested were significantly different between Southern and Northern Chinese. In the present study, this difference is not related to the subject’s gender, age or dietary habits; however, it may be due to the different genetic backgrounds of subjects of different ethnicities [31], [32]. Our study also showed that the serum concentrations of all food-specific IgGs except egg-specific IgG were positively associated with age. Previous studies have suggested that older people may be more likely than younger people to experience allergic reactions to foods [33]. It is not clear whether the higher levels of foods-specific IgGs in older persons indicates a higher probability of food allergies with increasing age. It is also unclear why egg-specific IgG concentrations were highest in the 20–34-year-old subjects and lowest in the 35–44-yearold subjects.

Previous studies have shown that abdominal discomforts self-attributed to food hypersensitivity is not related to eating habits or meal patterns [34]. Our study also showed that although the dietary frequencies of the 14 foods differed significantly in the demographic subgroups, only the dietary frequencies of chicken, egg, cow’s milk, rice and tomato were associated with the corresponding food-specific IgG serum levels after adjustment for demographic factors. The immunological mechanism responsible for the association of chicken, egg, cow’s milk, rice and tomato eating patterns with food-specific serum IgG concentrations is unclear, and further studies of this phenomenon are needed.

Previous studies have shown that chronic symptoms are closely related to food hypersensitivity [2], [6]. In our study, we found the food-specific concentrations of a few IgGs to be associated with a variety of chronic symptoms; the levels of some food-specific IgG were inversely associated with chronic symptoms, while the levels of other food-specific IgG were positively associated with chronic symptoms. Chronic symptoms were positively related to the serum levels of certain food-specific IgG. This may be a result of chronic inflammatory responses induced by elevated IgG or IgG subclasses in highly sensitive individuals [5]. For instance, gliadins and glutenins in wheat may increase specific IgG levels and may lead to chronic symptoms [35]. Our study also revealed negative associations between the level of rice-specific IgG and gastrointestinal symptoms, rhinitis, migraine, urticaria and eczema; such associations may result from different (protective or negative) roles of different IgG antibodies. Similarly, Tay et al [36] found that strong IgG responses to ovalbumin may be a normal physiological response to protein ingestion frequently shown in infancy, whereas up-regulated IgG responses that occur in peanut allergy may be indicative of a dysregulated immune response to peanut allergens. The normal physiological response reflected by the presence of food-specific IgG subclasses may be immunological tolerance due to the secretion of IL-10 by the activation of regulatory T cells upon prolonged exposure to food antigen [37].

The present study has several limitations. First, because food-specific IgG antibody testing is popular in China, we only tested for the presence of IgGs against commonly consumed foods, and did not measure levels of specific IgGs against pollen, fruits, nuts or food additives [1], [24], [25]. However, it should be kept in mind that self-reported chronic symptoms may result from the presence of IgGs specific for the latter foods and substances rather than from the presence of IgGs against the 14 tested food allergens. Second, in our study, the prevalence of chronic symptoms was determined by self-reporting of such symptoms on a questionnaire. The chronic symptoms reported by the patients in this study have not been confirmed by the adjudication of professional doctors. Thus, misclassification of chronic symptoms is possible, and the conclusions of the study regarding the associations of specific chronic symptoms with certain food-specific IgG levels should be regarded cautiously. Third, children and adolescents 0–17 years of age were not included in our study. It has been reported that the presence of food-specific IgGs in serum is more common in children and adolescents than in adults [15]. In children and adolescents, high levels of specific IgG are not significantly associated with clinical symptoms, and are likely to represent a protective mechanism that can result in immune tolerance to the corresponding foods later in life [38]. However, a similar protective role of food-specific IgG and IgG subclasses in adults has not been confirmed [15], [21]. Finally, the study does not provide an answer to the question of whether food-specific IgGs play a protective role or an allergic role in adults. The immunological mechanisms by which food-specific IgGs are induced are also unclear. A complete understanding of the importance of the presence of food-specific IgGs requires further accumulation of epidemiologic and experimental data.

In summary, the present study demonstrates for the first time that food-specific IgG serum concentrations are variable in both healthy and symptomatic Chinese adults. These findings raise awareness that demographic factors, types of food and the presence or absence of specific chronic symptoms should be considered before food elimination treatment based on IgG testing in patients with chronic symptoms is used in clinical practice. Further elucidation of the role played by food-specific IgGs in food allergies and tolerance should be an objective of future studies.

## Supporting Information

Figure S1 **Map of China with participating sites and numbers of subjects.** The middle line represents the boundary between southern China and northern China. The dots and triangles represent the participating sites in northern China and southern China, respectively.
(TIF)

Figure S2 **Distribution of serum food-specific IgG concentrations for 14 foods in subgroups according to sex.** The white and gray columns represent men and women, respectively. ^*^ See Figure 1.
(TIF)

Figure S3 **Distribution of serum food-specific IgG concentrations for 14 foods in subgroups according to geographic region.** The white and gray columns represent participants in South China and North China, respectively. ^*^ See Figure 1.
(TIF)

Figure S4 **Distribution of serum food-specific IgG concentrations for 14 foods according to age group.** CI, confidence interval.
(TIF)

Figure S5 **Differences in serum food-specific IgG concentrations in subjects with and without chronic symptoms.** ^*^ *P* value <0.05. CI, confidence interval.
(TIF)

Table S1 **Means and standard deviations of log-transformed food-specific IgG concentrations.** *The means and standard deviations of log-transformed food-specific IgG concentrations were calculated after the undetectable food-specific IgG concentrations were set as the half-value of the limit of detection. †The means and standard deviations of log-transformed food-specific IgG concentrations were calculated using maximum likelihood estimation.
(DOC)

Table S2 Correlation of food-specific IgG concentrations among 14 foods*. *The Pearson correlation was used to analyze possible correlations among the food-specific IgG concentrations for 14 foods. **Indicates that the correlation is significant at the 0.01 level (2-tailed).
(DOC)

Table S3 **Regular intake (≥3 times/week) of 14 foods in 5394 subjects (number (%)).**
(DOC)

## References

[1] Sicherer SH (2011) Epidemiology of food allergy. J Allergy Clin Immunol 127: 594–602. 21236480 10.1016/j.jaci.2010.11.044

[2] Sampson HA (2004) Update on food allergy. J Allergy Clin Immunol 113: 805–819; quiz 820. 10.1016/j.jaci.2004.03.014 15131561

[3] Penard-Morand C, Raherison C, Kopferschmitt C, Caillaud D, Lavaud F, et al (2005) Prevalence of food allergy and its relationship to asthma and allergic rhinitis in schoolchildren. Allergy 60: 1165–1171. 16076302 10.1111/j.1398-9995.2005.00860.x

[4] Atkinson W, Sheldon TA, Shaath N, Whorwell PJ (2004) Food elimination based on IgG antibodies in irritable bowel syndrome: a randomised controlled trial. Gut 53: 1459–1464. 15361495 10.1136/gut.2003.037697 PMC1774223

[5] Alpay K, Ertas M, Orhan EK, Ustay DK, Lieners C, et al (2010) Diet restriction in migraine, based on IgG against foods: a clinical double-blind, randomised, cross-over trial. Cephalalgia 30: 829–837. 20647174 10.1177/0333102410361404 PMC2899772

[6] Boyce JA, Assa’ad A, Burks AW, Jones SM, Sampson HA, et al (2010) Guidelines for the diagnosis and management of food allergy in the United States: report of the NIAID-sponsored expert panel. J Allergy Clin Immunol 126: S1–58. 21134576 10.1016/j.jaci.2010.10.007 PMC4241964

[7] Chafen JJ, Newberry SJ, Riedl MA, Bravata DM, Maglione M, et al (2010) Diagnosing and managing common food allergies: a systematic review. JAMA 303: 1848–1856. 20460624 10.1001/jama.2010.582

[8] Host A, Husby S, Gjesing B, Larsen JN, Lowenstein H (1992) Prospective estimation of IgG, IgG subclass and IgE antibodies to dietary proteins in infants with cow milk allergy. Levels of antibodies to whole milk protein, BLG and ovalbumin in relation to repeated milk challenge and clinical course of cow milk allergy. Allergy 47: 218–229. 1510234 10.1111/j.1398-9995.1992.tb00654.x

[9] Zar S, Benson MJ, Kumar D (2005) Food-specific serum IgG4 and IgE titers to common food antigens in irritable bowel syndrome. Am J Gastroenterol 100: 1550–1557. 15984980 10.1111/j.1572-0241.2005.41348.x

[10] Dreskin SC, Tripputi MT, Aubrey MT, Mustafa SS, Atkins D, et al (2010) Peanut-allergic subjects and their peanut-tolerant siblings have large differences in peanut-specific IgG that are independent of HLA class II. Clin Immunol 137: 366–373. 20850383 10.1016/j.clim.2010.08.009 PMC2976616

[11] Crowe SE, Perdue MH (1992) Gastrointestinal food hypersensitivity: basic mechanisms of pathophysiology. Gastroenterology 103: 1075–1095. 1499910 10.1016/0016-5085(92)90047-3

[12] Shreffler WG, Lencer DA, Bardina L, Sampson HA (2005) IgE and IgG4 epitope mapping by microarray immunoassay reveals the diversity of immune response to the peanut allergen, Ara h 2. J Allergy Clin Immunol 116: 893–899. 16210066 10.1016/j.jaci.2005.06.033

[13] Scott-Taylor TH, J OBH, Strobel S (2010) Correlation of allergen-specific IgG subclass antibodies and T lymphocyte cytokine responses in children with multiple food allergies. Pediatr Allergy Immunol 21: 935–944. 20444160 10.1111/j.1399-3038.2010.01025.x

[14] Johansson SG, Bieber T, Dahl R, Friedmann PS, Lanier BQ, et al (2004) Revised nomenclature for allergy for global use: report of the Nomenclature Review Committee of the World Allergy Organization, October 2003. J Allergy Clin Immunol 113: 832–836. 15131563 10.1016/j.jaci.2003.12.591

[15] Stapel SO, Asero R, Ballmer-Weber BK, Knol EF, Strobel S, et al (2008) Testing for IgG4 against foods is not recommended as a diagnostic tool: EAACI Task Force Report. Allergy 63: 793–796. 18489614 10.1111/j.1398-9995.2008.01705.x

[16] Ahrens B, Lopes de Oliveira LC, Schulz G, Borres MP, Niggemann B, et al (2010) The role of hen’s egg-specific IgE, IgG and IgG4 in the diagnostic procedure of hen’s egg allergy. Allergy 65: 1554–1557. 20608920 10.1111/j.1398-9995.2010.02429.x

[17] Hochwallner H, Schulmeister U, Swoboda I, Twaroch TE, Vogelsang H, et al (2011) Patients suffering from non-IgE-mediated cow’s milk protein intolerance cannot be diagnosed based on IgG subclass or IgA responses to milk allergens. Allergy 66: 1201–1207. 21575008 10.1111/j.1398-9995.2011.02635.x

[18] Hunter JO (2005) Food elimination in IBS: the case for IgG testing remains doubtful. Gut 54: 1203; author reply 1203. PMC1774875 16009694

[19] Pelsser LM, Frankena K, Toorman J, Savelkoul HF, Dubois AE, et al (2011) Effects of a restricted elimination diet on the behaviour of children with attention-deficit hyperactivity disorder (INCA study): a randomised controlled trial. Lancet 377: 494–503. 21296237 10.1016/S0140-6736(10)62227-1

[20] Zuo XL, Li YQ, Li WJ, Guo YT, Lu XF, et al (2007) Alterations of food antigen-specific serum immunoglobulins G and E antibodies in patients with irritable bowel syndrome and functional dyspepsia. Clinical and experimental allergy : journal of the British Society for Allergy and Clinical Immunology 37: 823–830. 17517095 10.1111/j.1365-2222.2007.02727.x

[21] Antico A, Pagani M, Vescovi PP, Bonadonna P, Senna G (2011) Food-Specific IgG4 Lack Diagnostic Value in Adult Patients with Chronic Urticaria and Other Suspected Allergy Skin Symptoms. Int Arch Allergy Immunol 155: 52–56. 21109748 10.1159/000318736

[22] Kanagawa Y, Matsumoto S, Koike S, Imamura T (2009) Association analysis of food allergens. Pediatr Allergy Immunol 20: 347–352. 19538355 10.1111/j.1399-3038.2008.00791.x

[23] Young E, Stoneham MD, Petruckevitch A, Barton J, Rona R (1994) A population study of food intolerance. Lancet 343: 1127–1130. 7910231 10.1016/s0140-6736(94)90234-8

[24] Schafer T, Bohler E, Ruhdorfer S, Weigl L, Wessner D, et al (2001) Epidemiology of food allergy/food intolerance in adults: associations with other manifestations of atopy. Allergy 56: 1172–1179. 11736746 10.1034/j.1398-9995.2001.00196.x

[25] Zuberbier T, Edenharter G, Worm M, Ehlers I, Reimann S, et al (2004) Prevalence of adverse reactions to food in Germany - a population study. Allergy 59: 338–345. 14982518 10.1046/j.1398-9995.2003.00403.x

[26] Harish Babu BN, Mahesh PA, Venkatesh YP (2008) A cross-sectional study on the prevalence of food allergy to eggplant (Solanum melongena L.) reveals female predominance. Clinical and experimental allergy : journal of the British Society for Allergy and Clinical Immunology 38: 1795–1802. 18681854 10.1111/j.1365-2222.2008.03076.x

[27] Poulos LM, Waters AM, Correll PK, Loblay RH, Marks GB (2007) Trends in hospitalizations for anaphylaxis, angioedema, and urticaria in Australia, 1993–1994 to 2004–2005. J Allergy Clin Immunol 120: 878–884. 17931562 10.1016/j.jaci.2007.07.040

[28] Sicherer SH, Munoz-Furlong A, Sampson HA (2003) Prevalence of peanut and tree nut allergy in the United States determined by means of a random digit dial telephone survey: a 5-year follow-up study. J Allergy Clin Immunol 112: 1203–1207. 14657884 10.1016/s0091-6749(03)02026-8

[29] Chen Y, Stewart P, Johansen H, McRae L, Taylor G (2003) Sex difference in hospitalization due to asthma in relation to age. J Clin Epidemiol 56: 180–187. 12654413 10.1016/s0895-4356(02)00593-0

[30] Zaitsu M, Narita S, Lambert KC, Grady JJ, Estes DM, et al (2007) Estradiol activates mast cells via a non-genomic estrogen receptor-alpha and calcium influx. Molecular immunology 44: 1977–1985. 17084457 10.1016/j.molimm.2006.09.030 PMC2603032

[31] Hong X, Wang G, Liu X, Kumar R, Tsai HJ, et al.. (2011) Gene polymorphisms, breast-feeding, and development of food sensitization in early childhood. J Allergy Clin Immunol 128: 374–381 e372. 10.1016/j.jaci.2011.05.007 PMC3149737 21689850

[32] Campos Alberto EJ, Shimojo N, Suzuki Y, Mashimo Y, Arima T, et al (2008) IL-10 gene polymorphism, but not TGF-beta1 gene polymorphisms, is associated with food allergy in a Japanese population. Pediatr Allergy Immunol 19: 716–721. 18208460 10.1111/j.1399-3038.2007.00709.x

[33] Harduar-Morano L, Simon MR, Watkins S, Blackmore C (2011) A population-based epidemiologic study of emergency department visits for anaphylaxis in Florida. The Journal of allergy and clinical immunology 128: 594–600 e591. 10.1016/j.jaci.2011.04.049 PMC3970843 21714994

[34] Carvalho RV, Lorena SL, Almeida JR, Mesquita MA (2010) Food intolerance, diet composition, and eating patterns in functional dyspepsia patients. Dig Dis Sci 55: 60–65. 19160046 10.1007/s10620-008-0698-8

[35] Battais F, Pineau F, Popineau Y, Aparicio C, Kanny G, et al (2003) Food allergy to wheat: identification of immunogloglin E and immunoglobulin G-binding proteins with sequential extracts and purified proteins from wheat flour. Clin Exp Allergy 33: 962–970. 12859454 10.1046/j.1365-2222.2003.01592.x

[36] Tay SS, Clark AT, Deighton J, King Y, Ewan PW (2007) Patterns of immunoglobulin G responses to egg and peanut allergens are distinct: ovalbumin-specific immunoglobulin responses are ubiquitous, but peanut-specific immunoglobulin responses are up-regulated in peanut allergy. Clin Exp Allergy 37: 1512–1518. 17883730 10.1111/j.1365-2222.2007.02802.x

[37] Satoguina JS, Weyand E, Larbi J, Hoerauf A (2005) T regulatory-1 cells induce IgG4 production by B cells: role of IL-10. J Immunol 174: 4718–4726. 15814696 10.4049/jimmunol.174.8.4718

[38] Skripak JM, Nash SD, Rowley H, Brereton NH, Oh S, et al (2008) A randomized, double-blind, placebo-controlled study of milk oral immunotherapy for cow’s milk allergy. J Allergy Clin Immunol 122: 1154–1160. 18951617 10.1016/j.jaci.2008.09.030 PMC3764488

