# Supplementary figures and images for: Variable Food-Specific IgG Antibody Levels in Healthy and Symptomatic Chinese Adults
Source: PLoS One. 2013 Jan 3;8(1):e53612. doi: 10.1371/journal.pone.0053612 (PMC3536737; doi:10.1371/journal.pone.0053612)

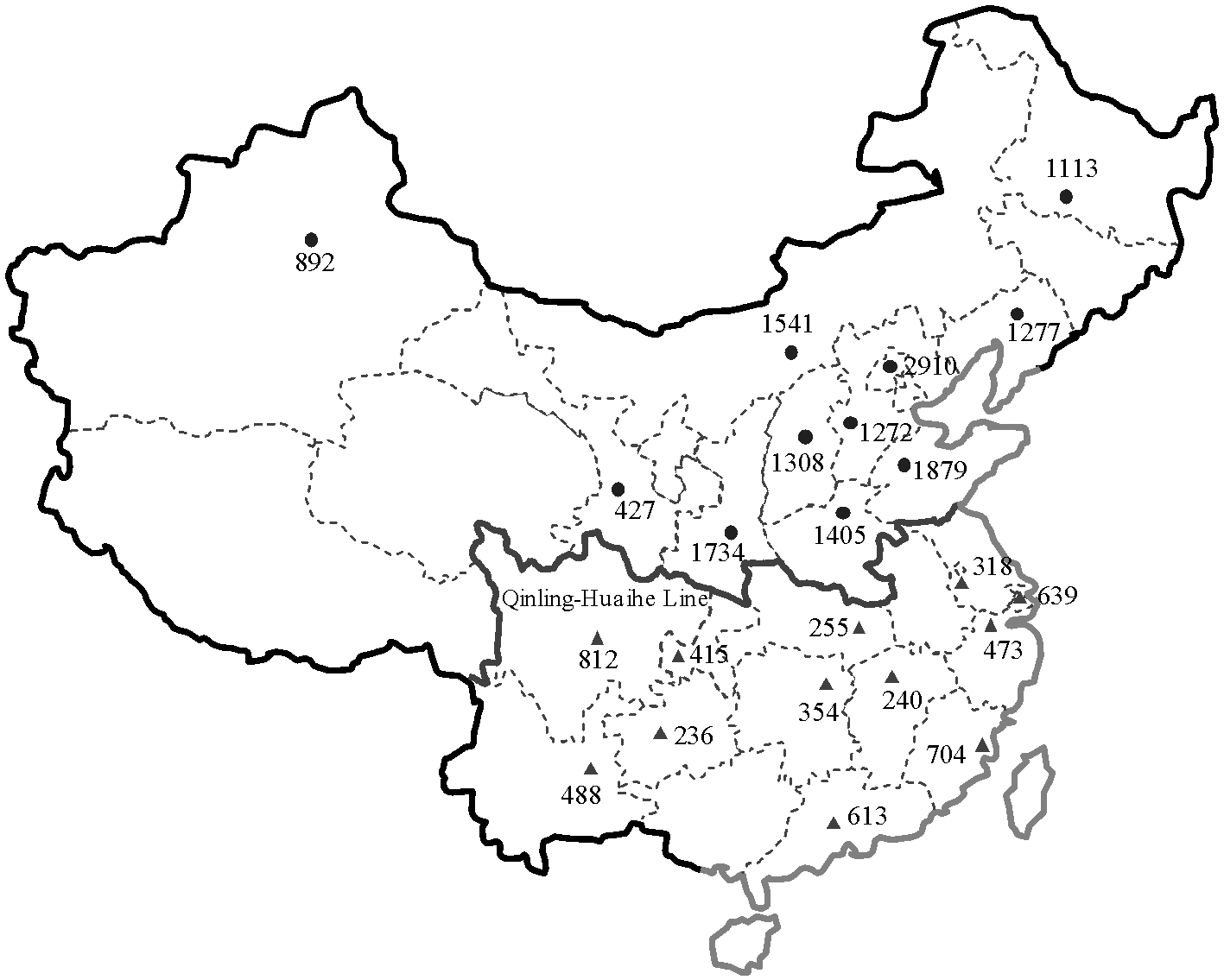

Supplement: Figure S1 — Map of China with participating sites and numbers of subjects. The middle line represents the boundary between southern China and northern China. The dots and triangles represent the participating sites in northern China and southern China, respectively. (TIF) [file pone.0053612.s001.tif]

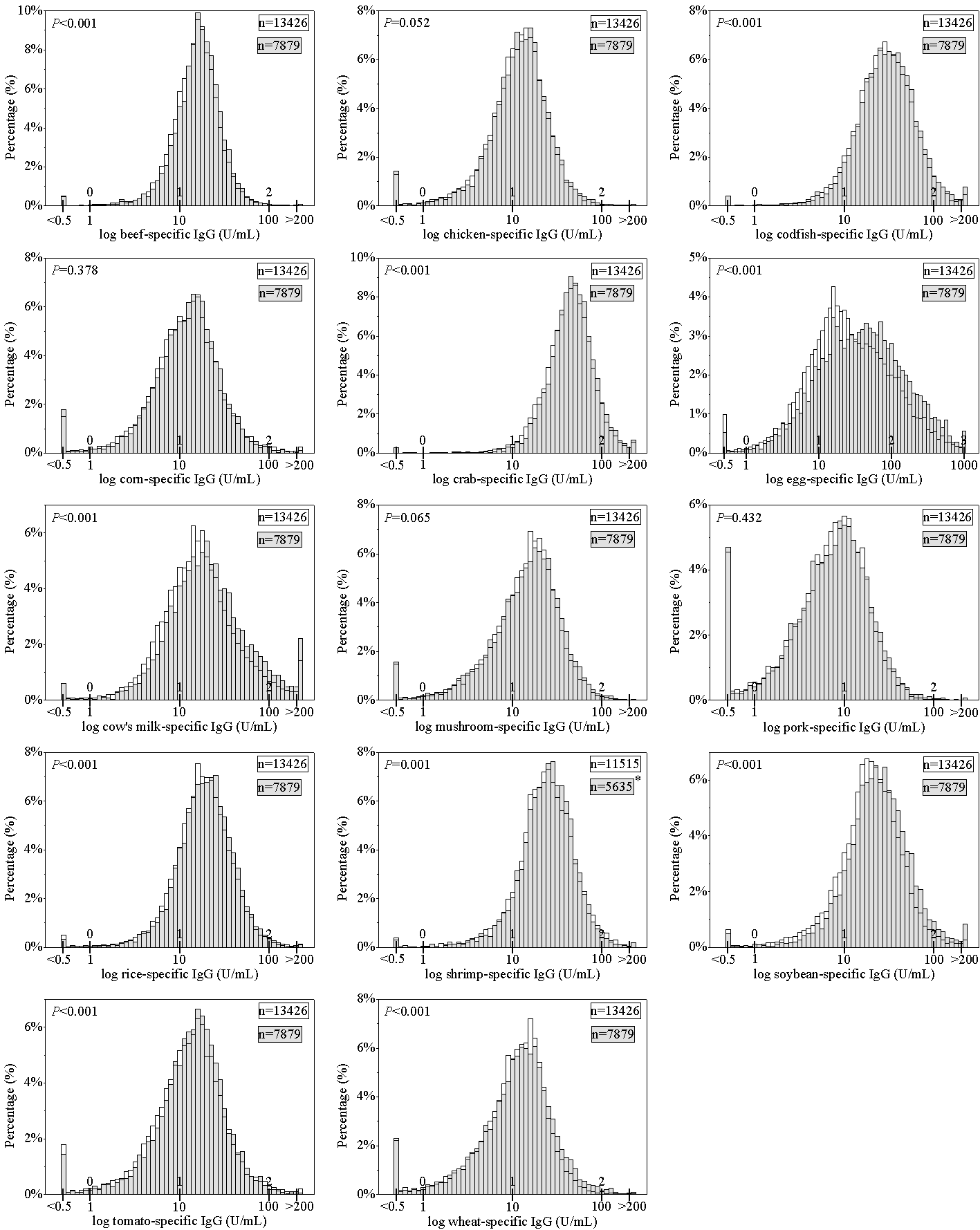

Supplement: Figure S2 — Distribution of serum food-specific IgG concentrations for 14 foods in subgroups according to sex. The white and gray columns represent men and women, respectively. * See Figure 1. (TIF) [file pone.0053612.s002.tif]

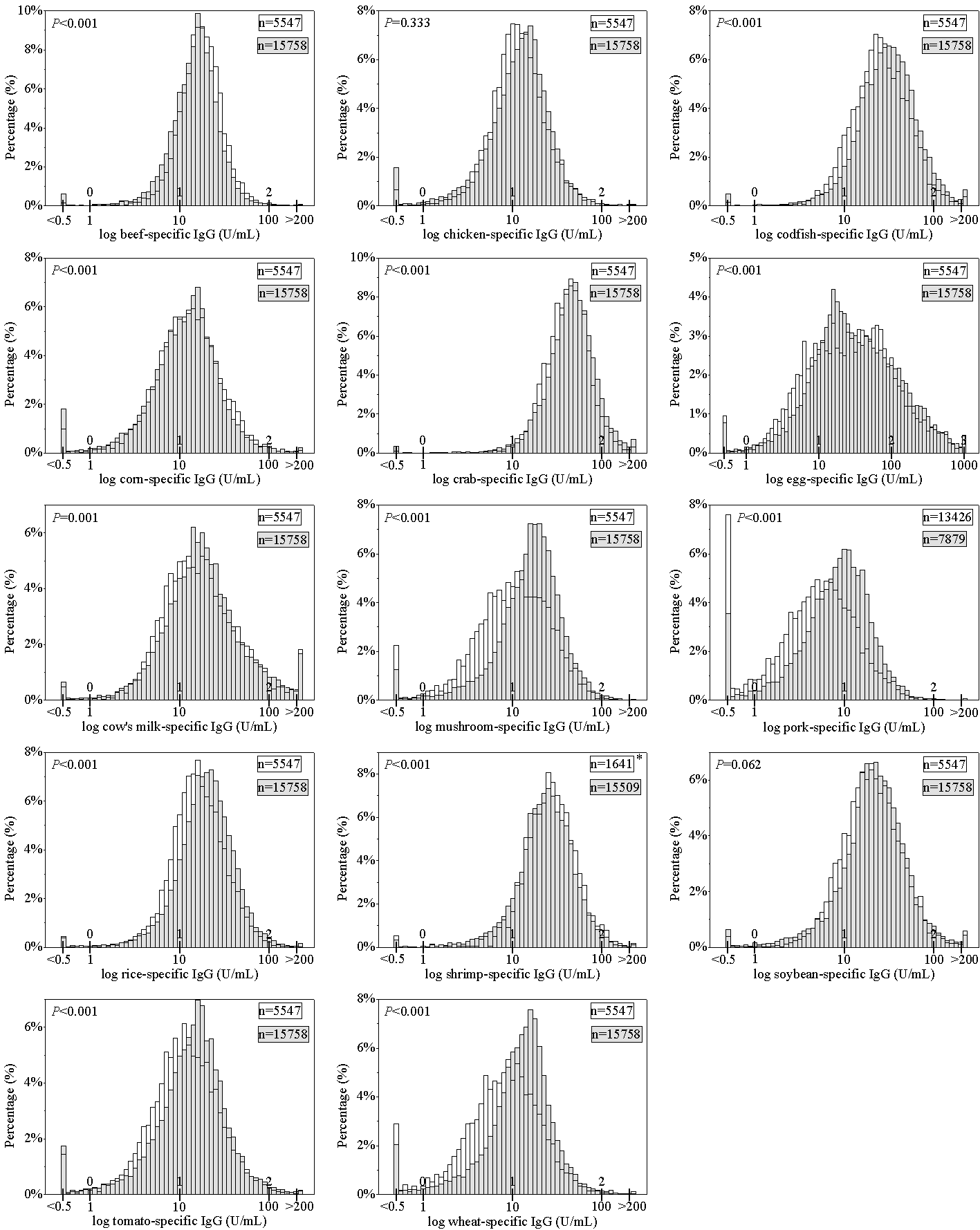

Supplement: Figure S3 — Distribution of serum food-specific IgG concentrations for 14 foods in subgroups according to geographic region. The white and gray columns represent participants in South China and North China, respectively. * See Figure 1. (TIF) [file pone.0053612.s003.tif]

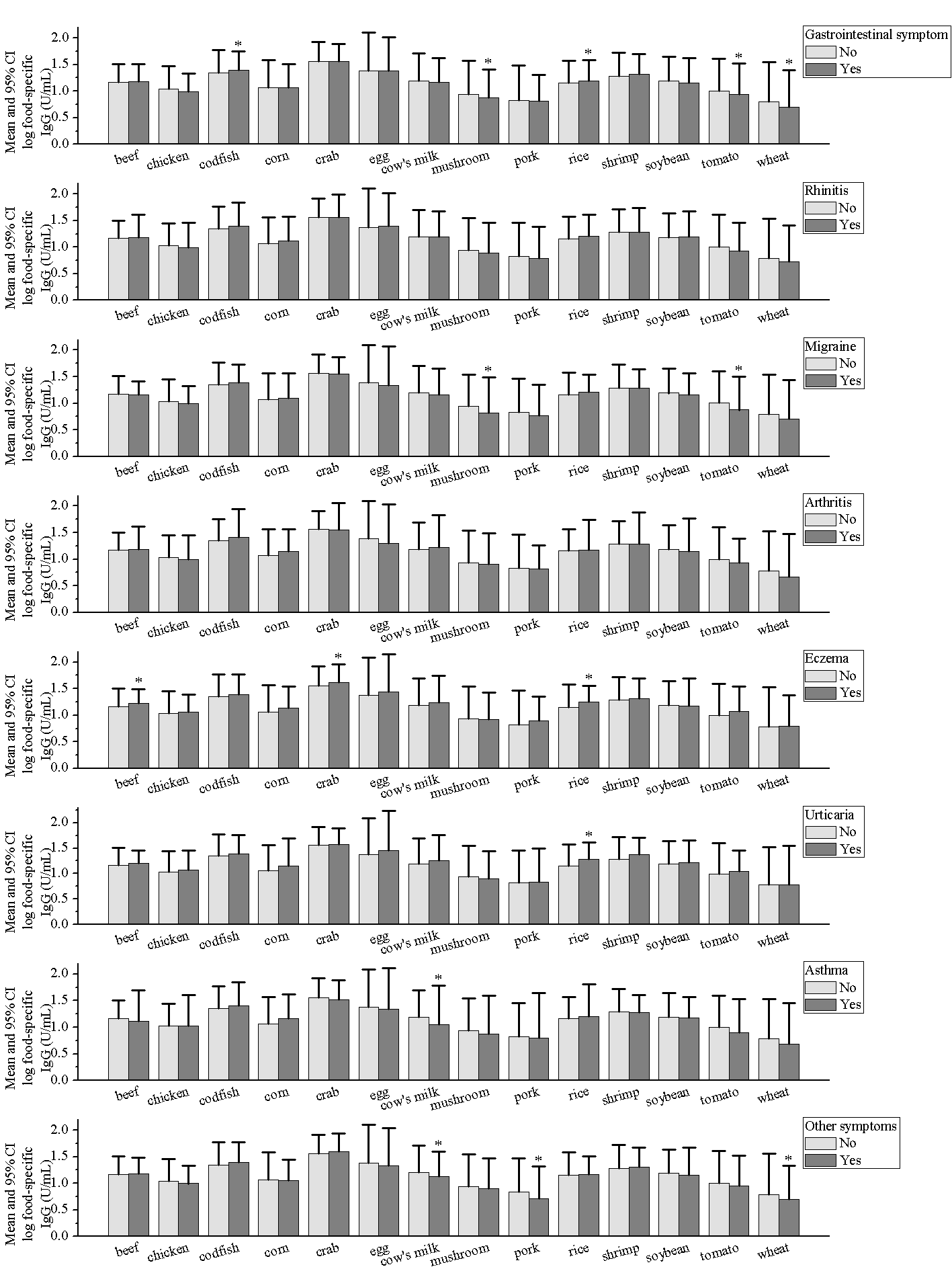

Supplement: Figure S5 — Differences in serum food-specific IgG concentrations in subjects with and without chronic symptoms. * P value <0.05. CI, confidence interval. (TIF) [file pone.0053612.s005.tif]
