# Supplementary material for: Variable Food-Specific IgG Antibody Levels in Healthy and Symptomatic Chinese Adults
Source: PLoS One. 2013 Jan 3;8(1):e53612. doi: 10.1371/journal.pone.0053612 (PMC3536737; doi:10.1371/journal.pone.0053612)
Supplement: Table S1 — Means and standard deviations of log-transformed food-specific IgG concentrations. *The means and standard deviations of log-transformed food-specific IgG concentrations were calculated after the undetectable food-specific IgG concentrations were set as the half-value of the limit of detection. †The means and standard deviations of log-transformed food-specific IgG concentrations were calculated using maximum likelihood estimation. (DOC) [file pone.0053612.s006.doc]

**Table S1. M**eans and standard deviations of log-transformed food-specific IgG concentrations

| Type | Half of the limit of detection* | |  | Maximum likelihood estimation† | |
| --- | --- | --- | --- | --- | --- |
|  | Mean | Standard deviation |  | Mean | Standard deviation |
| Beef | 1.146 | 0.346 |  | 1.153 | 0.456 |
| Chicken | 1.011 | 0.458 |  | 1.021 | 0.595 |
| Coldfish | 1.420 | 0.395 |  | 1.428 | 0.544 |
| Corn | 1.009 | 0.517 |  | 1.024 | 0.667 |
| Crab | 1.603 | 0.338 |  | 1.609 | 0.452 |
| Egg | 1.442 | 0.644 |  | 1.452 | 0.931 |
| Mushroom | 1.082 | 0.511 |  | 1.093 | 0.667 |
| Milk | 1.213 | 0.484 |  | 1.220 | 0.696 |
| Pork | 0.737 | 0.675 |  | 0.761 | 0.819 |
| Rice | 1.226 | 0.376 |  | 1.232 | 0.520 |
| Shrimp | 1.333 | 0.373 |  | 1.339 | 0.520 |
| Soybean | 1.276 | 0.427 |  | 1.283 | 0.597 |
| Tomato | 1.045 | 0.527 |  | 1.060 | 0.689 |
| Wheat | 0.953 | 0.555 |  | 0.972 | 0.711 |

*The means and standard deviations of log-transformed food-specific IgG concentrations were calculated after the undetectable food-specific IgG concentrations were set as the half-value of the limit of detection.

†The means and standard deviations of log-transformed food-specific IgG concentrations were calculated using maximum likelihood estimation.
