# Supplementary material for: Variable Food-Specific IgG Antibody Levels in Healthy and Symptomatic Chinese Adults
Source: PLoS One. 2013 Jan 3;8(1):e53612. doi: 10.1371/journal.pone.0053612 (PMC3536737; doi:10.1371/journal.pone.0053612)
Supplement: Table S2 — Correlation of food-specific IgG concentrations among 14 foods*. *The Pearson correlation was used to analyze possible correlations among the food-specific IgG concentrations for 14 foods. **Indicates that the correlation is significant at the 0.01 level (2-tailed). (DOC) [file pone.0053612.s007.doc]

**Table S2. Correlation of food-specific IgG concentrations among 14 foods***.

|  | beef | chicken | codfish | corn | crab | egg | mushroom | cow's milk | pork | rice | shrimp | soybean | tomato | wheat |
| --- | --- | --- | --- | --- | --- | --- | --- | --- | --- | --- | --- | --- | --- | --- |
| beef |  | 0.691^**^ | 0.502^**^ | 0.527^**^ | 0.521^**^ | 0.232^**^ | 0.442^**^ | 0.332^**^ | 0.418^**^ | 0.439^**^ | 0.444^**^ | 0.349^**^ | 0.506^**^ | 0.317^**^ |
| chicken |  |  | 0.492^**^ | 0.556^**^ | 0.441^**^ | 0.277^**^ | 0.486^**^ | 0.362^**^ | 0.502^**^ | 0.469^**^ | 0.430^**^ | 0.378^**^ | 0.504^**^ | 0.413^**^ |
| codfish |  |  |  | 0.401^**^ | 0.561^**^ | 0.218^**^ | 0.438^**^ | 0.313^**^ | 0.355^**^ | 0.407^**^ | 0.406^**^ | 0.365^**^ | 0.395^**^ | 0.296^**^ |
| corn |  |  |  |  | 0.390^**^ | 0.252^**^ | 0.433^**^ | 0.361^**^ | 0.435^**^ | 0.481^**^ | 0.373^**^ | 0.373^**^ | 0.525^**^ | 0.411^**^ |
| crab |  |  |  |  |  | 0.185^**^ | 0.419^**^ | 0.252^**^ | 0.261^**^ | 0.378^**^ | 0.444^**^ | 0.345^**^ | 0.384^**^ | 0.218^**^ |
| egg |  |  |  |  |  |  | 0.246^**^ | 0.346^**^ | 0.217^**^ | 0.320^**^ | 0.249^**^ | 0.295^**^ | 0.291^**^ | 0.336^**^ |
| mushroom |  |  |  |  |  |  |  | 0.307^**^ | 0.453^**^ | 0.449^**^ | 0.409^**^ | 0.368^**^ | 0.514^**^ | 0.518^**^ |
| cow' milk |  |  |  |  |  |  |  |  | 0.341^**^ | 0.447^**^ | 0.379^**^ | 0.425^**^ | 0.381^**^ | 0.426^**^ |
| pork |  |  |  |  |  |  |  |  |  | 0.433^**^ | 0.358^**^ | 0.295^**^ | 0.465^**^ | 0.450^**^ |
| rice |  |  |  |  |  |  |  |  |  |  | 0.551^**^ | 0.516^**^ | 0.491^**^ | 0.530^**^ |
| shrimp |  |  |  |  |  |  |  |  |  |  |  | 0.491^**^ | 0.427^**^ | 0.434^**^ |
| soybean |  |  |  |  |  |  |  |  |  |  |  |  | 0.407^**^ | 0.457^**^ |
| tomato |  |  |  |  |  |  |  |  |  |  |  |  |  | 0.553^**^ |
| wheat |  |  |  |  |  |  |  |  |  |  |  |  |  |  |

*The Pearson correlation was used to analyze possible correlations among the food-specific IgG concentrations for 14 foods.

**Indicates that the correlation is significant at the 0.01 level (2-tailed).
