# Supplementary material for: Variable Food-Specific IgG Antibody Levels in Healthy and Symptomatic Chinese Adults
Source: PLoS One. 2013 Jan 3;8(1):e53612. doi: 10.1371/journal.pone.0053612 (PMC3536737; doi:10.1371/journal.pone.0053612)
Supplement: Table S3 — Regular intake (≥3 times/week) of 14 foods in 5394 subjects (number (%)). (DOC) [file pone.0053612.s008.doc]

**Table S3**. Regular intake (≥3 times/week) of 14 foods in 5394 subjects (number (%))

| Type | Overall  n=5394 | Gender | | |  | Region | | |  | Age | | | | | |
| --- | --- | --- | --- | --- | --- | --- | --- | --- | --- | --- | --- | --- | --- | --- | --- |
|  |  | Men  n=3659 | Women  n=1735 | *P* value |  | South  n=1231 | North  n=4163 | *P* value |  | 18-34  n=462 | 35-44  n=1751 | 45-54  n=2025 | 55-64 n=733 | ≥65  n=423 | *P* for trend |
| Egg | 3029 (56.2) | 2039 (55.7) | 990 (57.1) | 0.356 |  | 691 (56.1) | 2338 (56.2) | 0.986 |  | 237 (51.3) | 950 (54.3) | 1148 (56.7) | 421 (57.4) | 273 (64.5) | <0.001 |
| Crab | 370  (6.9) | 238  (6.5) | 132  (7.6) | 0.134 |  | 215 (17.5) | 155  (3.7) | <0.001 |  | 43  (9.3) | 125  (7.1) | 111  (5.5) | 53  (7.2) | 38  (9.0) | 0.723 |
| Cow’s milk | 2572 (47.7) | 1589 (43.4) | 983 (56.7) | <0.001 |  | 484 (39.3) | 2088 (50.2) | <0.001 |  | 224 (48.5) | 733 (41.9) | 939 (46.4) | 387  (52.8) | 289 (68.3) | <0.001 |
| Shrimp | 832 (15.4) | 567 (15.5) | 265 (15.3) | 0.833 |  | 315 (25.6) | 517 (12.4) | <0.001 |  | 99 (21.4) | 257 (14.7) | 311 (15.4) | 112  (15.3) | 53 (12.5) | 0.014 |
| Codfish | 990 (18.4) | 708 (19.3) | 282 (16.3) | 0.006 |  | 401 (32.6) | 589 (14.1) | <0.001 |  | 85 (18.4) | 312 (17.8) | 394 (19.5) | 133  (18.1) | 66 (15.6) | 0.591 |
| Soybean | 1735 (32.2) | 1118 (30.6) | 617 (35.6) | <0.001 |  | 426 (34.6) | 1309 (31.4) | 0.037 |  | 121 (26.2) | 499 (28.5) | 660 (32.6) | 277  (37.8) | 178 (42.1) | <0.001 |
| Corn | 1068 (19.8) | 628 (17.2) | 440 (25.4) | <0.001 |  | 258 (21.0) | 810 (19.5) | 0.245 |  | 94 (20.3) | 245 (14.0) | 420 (20.7) | 177  (24.1) | 132 (31.2) | <0.001 |
| Tomato | 2215 (41.1) | 1418 (38.8) | 797 (45.9) | <0.001 |  | 357 (29.0) | 1858 (44.6) | <0.001 |  | 167 (36.1) | 656 (37.5) | 855 (42.2) | 297  (40.5) | 240 (56.7) | <0.001 |
| Chicken | 986 (18.3) | 737 (20.1) | 249 (14.4) | <0.001 |  | 241 (19.6) | 745 (17.9) | 0.180 |  | 102 (22.1) | 320 (18.3) | 386 (19.1) | 122  (16.6) | 56 (13.2) | 0.002 |
| Beef | 1204 (22.3) | 936 (25.6) | 268 (15.4) | <0.001 |  | 232 (18.8) | 972 (23.3) | 0.001 |  | 122 (26.4) | 403 (23.0) | 498 (24.6) | 122  (16.6) | 59 (13.9) | <0.001 |
| Rice | 4520 (83.8) | 2979 (81.4) | 1541 (88.8) | <0.001 |  | 1171 (95.1) | 3349 (80.4) | <0.001 |  | 417 (90.3) | 1474 (84.2) | 1627 (80.3) | 630  (85.9) | 372 (87.9) | 0.579 |
| Mushroom | 1025 (19.0) | 668 (18.3) | 357 (20.6) | 0.042 |  | 322 (26.2) | 703 (16.9) | <0.001 |  | 83 (18.0) | 314 (17.9) | 389 (19.2) | 152  (20.7) | 87 (20.6) | 0.069 |
| Wheat | 3339 (61.9) | 2273 (62.1) | 1066 (61.4) | 0.631 |  | 436 (35.4) | 2903 (69.7) | <0.001 |  | 231 (50.0) | 1009 (57.6) | 1326 (65.5) | 478  (65.2) | 295 (69.7) | <0.001 |
| Pork | 3781 (70.1) | 2618 (71.5) | 1163 (67.0) | 0.001 |  | 1022 (83.0) | 2759 (66.3) | <0.001 |  | 346 (74.9) | 1291 (73.7) | 1402 (69.2) | 483  (65.9) | 259 (61.2) | <0.001 |
